# Supplementary material for: Preparation and purification of mono-ubiquitinated proteins using Avi-tagged ubiquitin
Source: PLoS One. 2020 Feb 24;15(2):e0229000. doi: 10.1371/journal.pone.0229000 (PMC7039436; doi:10.1371/journal.pone.0229000)
Supplement: S1 Fig — (A) Coomassie stained 3–8% Tris-acetate gel of Flag- and Talon-affinity purification of human FANCI:FANCD2 complex, and Western blot of Talon-purified FANCI:FANCD2 complex. (B) Coomassie stained SDS-PAGE gel revealing that human FANCI runs higher than Xenopus FANCI in the FANCI:FANCD2 complex. (C) Mono-ubiquitination of human and Xenopus FANCI:FANCD2 complex in a reaction containing recombinant FA core complex proteins at 25°C for 90 min. (PDF) [file pone.0229000.s001.pdf]

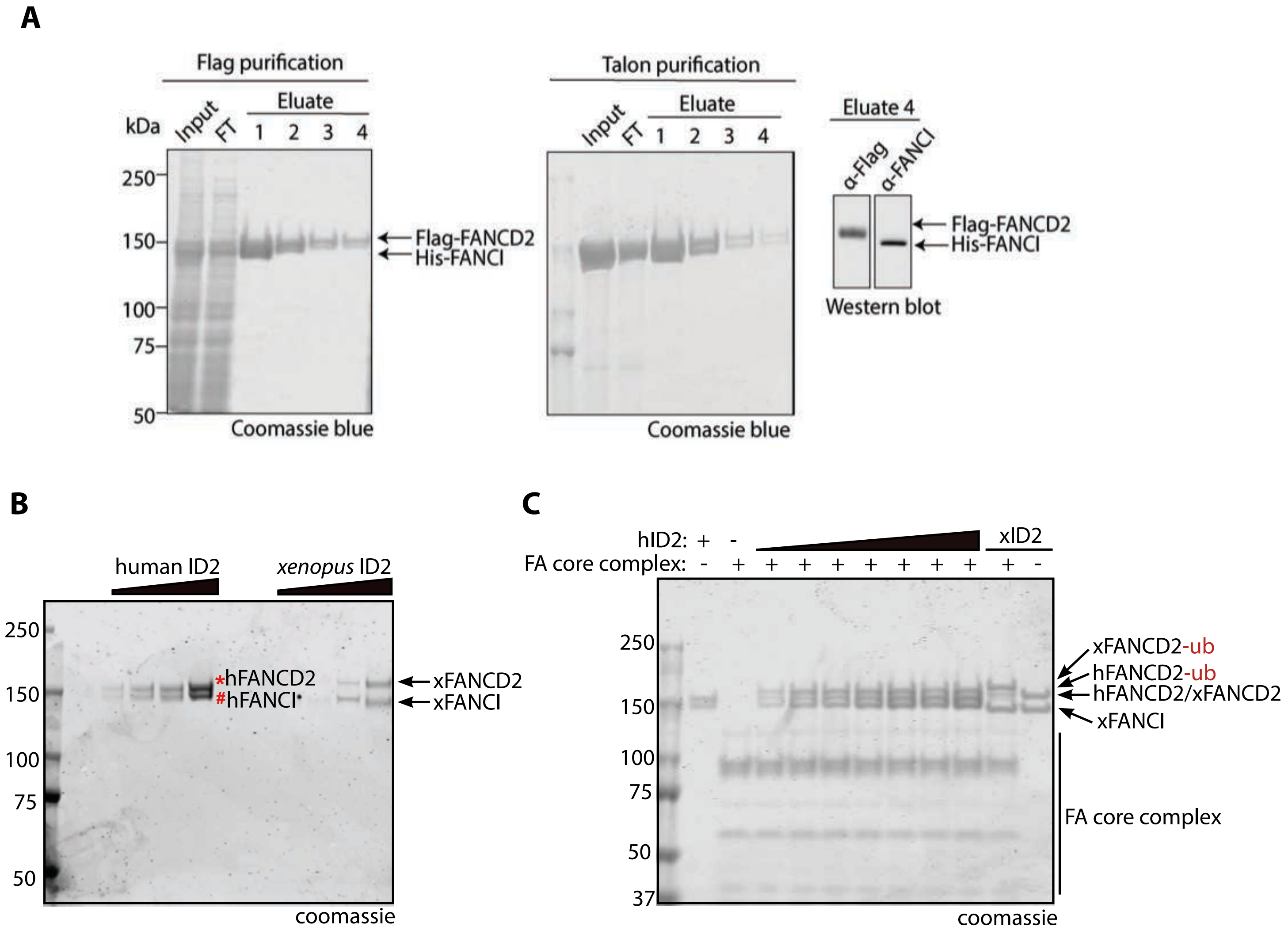

Supplementary Figure 1. Purification and mono-ubiquitination of human FANCI:FANCD2 complex. (A) Coomassie stained 3-8% Tris-acetate gel of Flag- and Talon-affinity purification of human FANCI:FANCD2 complex, and Western blot of Talon-purified FANCI:FANCD2 complex. **(B)** Coomassie stained SDS-PAGE gel revealing that human FANCI runs higher than *Xenopus* FANCI in the FANCI:FANCD2 complex. **(C)** Mono-ubiquitination of human and *Xenopus* FANCI:FANCD2 complex in a reaction containing recombinant FA core complex proteins at 25 °C for 90 min.
