# Supplementary material for: Preparation and purification of mono-ubiquitinated proteins using Avi-tagged ubiquitin
Source: PLoS One. 2020 Feb 24;15(2):e0229000. doi: 10.1371/journal.pone.0229000 (PMC7039436; doi:10.1371/journal.pone.0229000)
Supplement: S2 Fig — (A) Coomassie stained SDS-PAGE showing reconstitution of mono-ubiquitinated nucleosomes. Lane 1 shows 147 bp dsDNA based on Widom 601 sequence in the absence of nucleosome (Widom 147), lane 2 shows mono-ubiquitination reaction containing nucleosomes without ATP (nucleosomes) and lane 3 shows mono-ubiquitination reaction containing nucleosomes, BRCA1Δexon11:BARD1 complex, Avi-biotin-ubiquitin, UBE1, UBCH5C and ATP (ubiquitinated nucleosomes). (B) 6% native PAGE gel containing the same reactions in (A) stained with SYBR gold nucleic acid stain showing reconstitution of nucleosomes (lanes 2–3). (C) Western blot of the 6% P AGE from (B) probed against Streptavidin antibody showing that the reconstituted nucleosomes were ubiquitinated (lane 3). (PDF) [file pone.0229000.s002.pdf]

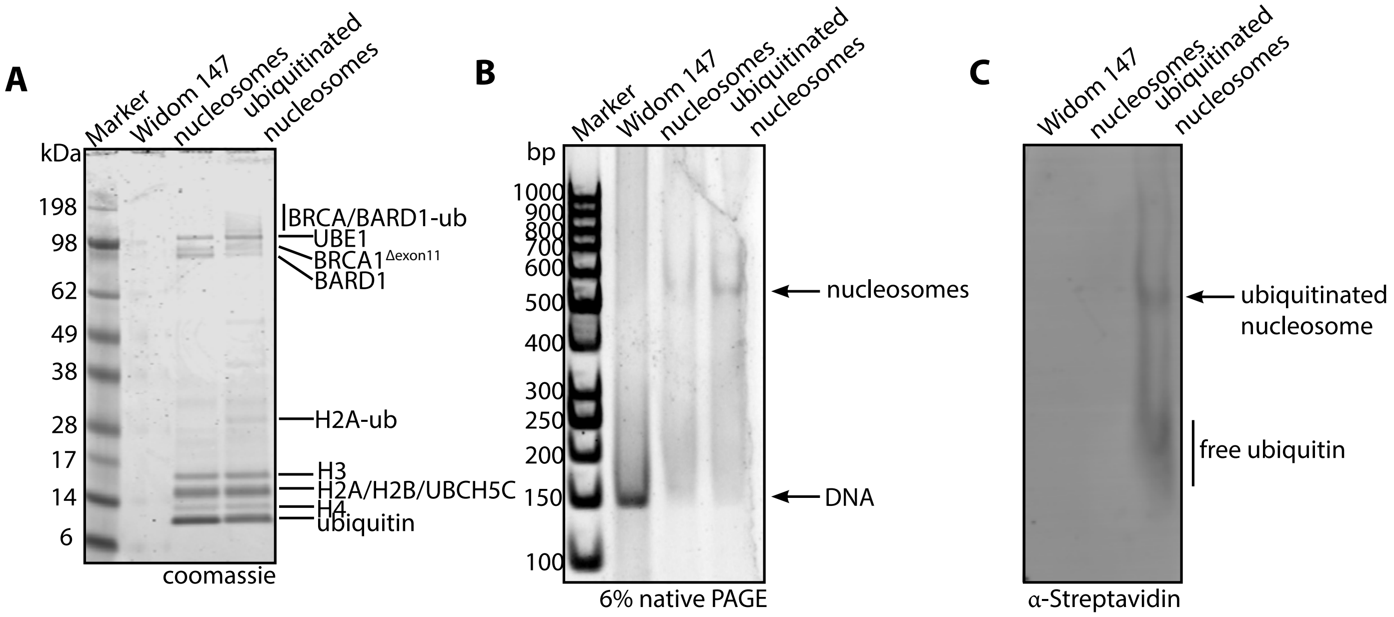

Supplementary Figure 2. Biochemical reconstitution of mono-ubiquitinated nucleosomes. (A) Coomassie stained SDS-PAGE showing reconstitution of mono-ubiquitinated nucleosomes. Lane 1 shows 147 bp dsDNA based on Widom 601 sequence in the absence of nucleosome (Widom 147), lane 2 shows mono-ubiquitination reaction containing nucleosomes without ATP (nucleosomes) and lane 3 shows mono-ubiquitination reaction containing nucleosomes, BRCA1$^{\Delta exon11}$:BARD1 complex, Avi-biotin-ubiquitin, UBE1, UBCH5C and ATP (ubiquitinated nucleosomes). **(B)** 6% native PAGE gel containing the same reactions in (A) stained with SYBR gold nucleic acid stain showing reconstitution of nucleosomes (lanes 2-3). **(C)** Western blot of the 6% PAGE from (B) probed against Streptavidin antibody showing that the reconstituted nucleosomes were ubiquitinated (lane 3).
