# Supplementary material for: Preparation and purification of mono-ubiquitinated proteins using Avi-tagged ubiquitin
Source: PLoS One. 2020 Feb 24;15(2):e0229000. doi: 10.1371/journal.pone.0229000 (PMC7039436; doi:10.1371/journal.pone.0229000)
Supplement: S3 Fig — (A-C) A gel band containing ubiquitinated FANCI, FANCD2 and PCNA were processed and in-gel digested with trypsin and analyzed by mass spectrometry. The tandem mass (M S/M S) spectrum of mono-ubiquitinated peptides were derived by collision-induced dissociation of the (M+H)+ precursor, m/z as indicated. Fragment ions in the spectrum represent mainly single-event preferential cleavage of the peptide bonds resulting in the sequence information recorded simultaneously from both the N- and C-termini (b- and y-type ions, respectively) of the peptide. Data analysis was performed using M ascot (35) and the data was searched against the Uniprot database (downloaded 0606/2013). Searches were conducted with trypsin, the precursor ion tolerance was set to 10 ppm and the fragment ion tolerance was set to 0.2 Da. Variable modifications include oxidation on methionine (+15.995 Da), carbamidomethyl (+57.021 Da) on cysteine, and ubiquitination on glycine/glycine (+114.043 Da). The maximum missed cleavages were set to 2. All search results were evaluated by MudPIT scoring for false discovery rate (FDR) evaluation of the identified peptides (36) and peptides identifications were filtered to a FDR of 5%. Mass spectra verified that the purified FANCI is mono-ubiquitinated at lysine 523 (A), FANCD2 is mono-ubiquitinated at lysine 561 (B), and PCNA is mono-ubiquitinated at lysine 167 (C). (D) Summary of mass spectrometry results of mono-ubiquitinated BRCA1:BARD1. (PDF) [file pone.0229000.s003.pdf]

A

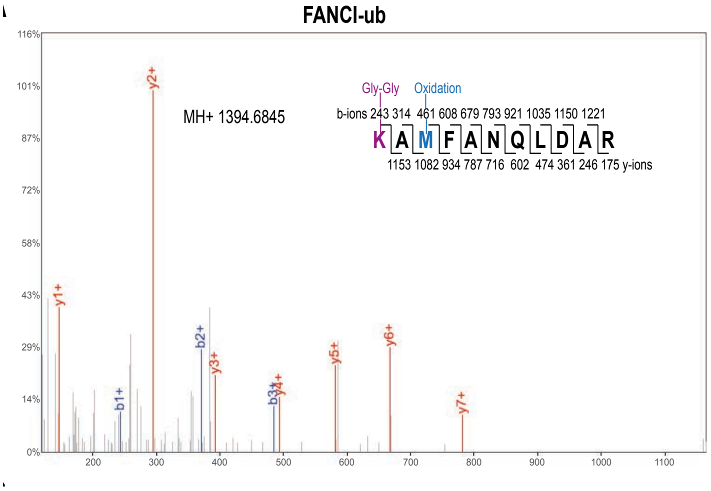

B

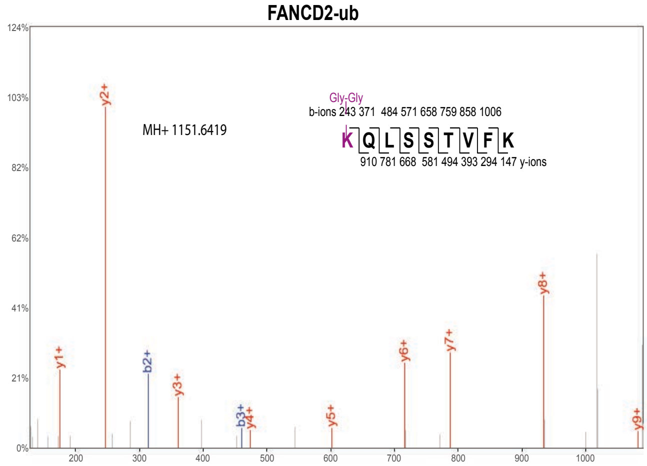

C

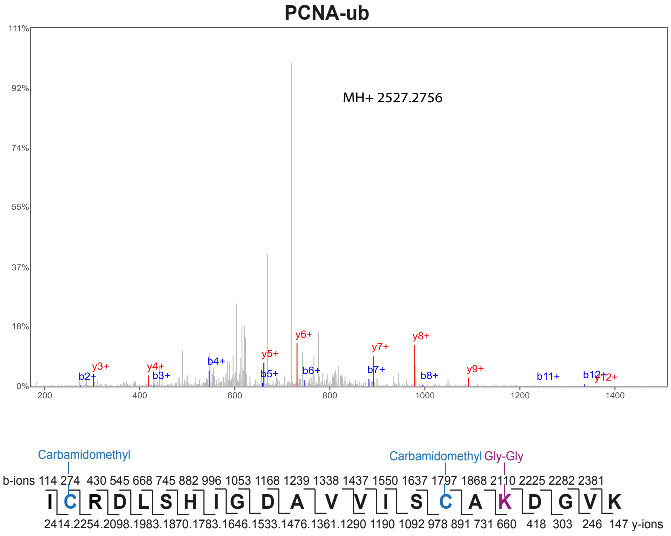

D

Protein	No. peptides	% coverage	Ubiquitinated residues
BRCA1$^{\Delta exon11}$	49	14	K223, K679, K731, K739, K748, K1487, K1489, K1476, K1500, K1606, K1601, K1636
BARD1	83	44	K46, K124, K139, K160, K171, K172, K232, K379, K384, K503

Supplementary Figure 3. Mass spectrometry results of mono-ubiquitinated FANCI, FANCD2, PCNA, BRCA1 and BARD1. (A-C) A gel band containing ubiquitinated FANCI, FANCD2 and PCNA were processed and in-gel digested with trypsin and analyzed by mass spectrometry. The tandem mass (MS/MS) spectrum of mono-ubiquitinated peptides were derived by collision-induced dissociation of the (M+H)+ precursor, m/z as indicated. Fragment ions in the spectrum represent mainly single-event preferential cleavage of the peptide bonds resulting in the sequence information recorded simultaneously from both the N- and C-termini (b- and y-type ions, respectively) of the peptide. Data analysis was performed using Mascot (35) and the data was searched against the Uniprot database (downloaded 0606/2013). Searches were conducted with trypsin, the precursor ion tolerance was set to 10 ppm and the fragment ion tolerance was set to 0.2 Da. Variable modifications include oxidation on methionine (+15.995 Da), carbamidomethyl (+57.021 Da) on cysteine, and ubiquitination on glycine/glycine (+114.043 Da). The maximum missed cleavages were set to 2. All search results were evaluated by MudPIT scoring for false discovery rate (FDR) evaluation of the identified peptides (36) and peptides identifications were filtered to a FDR of 5%. Mass spectra verified that the purified FANCI is mono-ubiquitinated at lysine 523 **(A)**, FANCD2 is mono-ubiquitinated at lysine 561 **(B)**, and PCNA is mono-ubiquitinated at lysine 167 **(C)**. **(D)** Summary of mass spectrometry results of mono-ubiquitinated BRCA1:BARD1.
