# Supplementary material for: Preparation and purification of mono-ubiquitinated proteins using Avi-tagged ubiquitin
Source: PLoS One. 2020 Feb 24;15(2):e0229000. doi: 10.1371/journal.pone.0229000 (PMC7039436; doi:10.1371/journal.pone.0229000)
Supplement: S4 Fig — Original source images for all data obtained by electrophoretic separation: Coomassie stained SDS-PAGE and western blots. (PDF) [file pone.0229000.s004.pdf]

Figure 1B

Coomassie stain

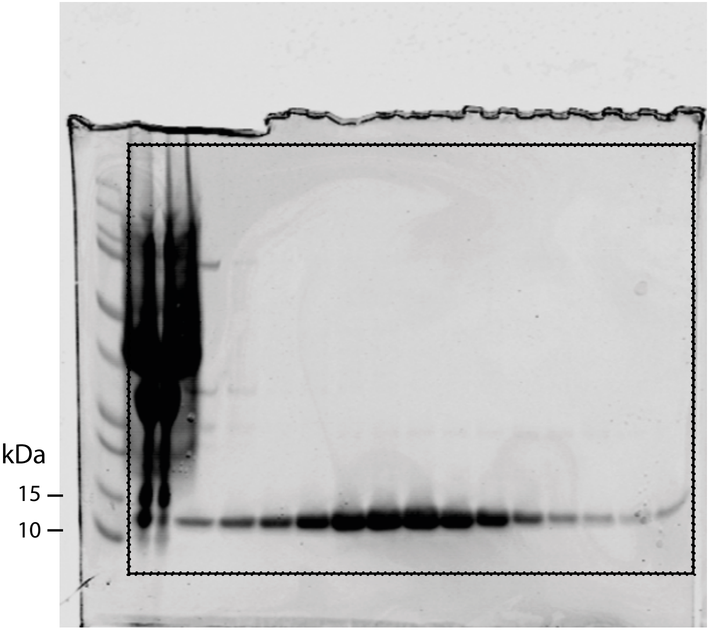

Figure 1C

Coomassie stain

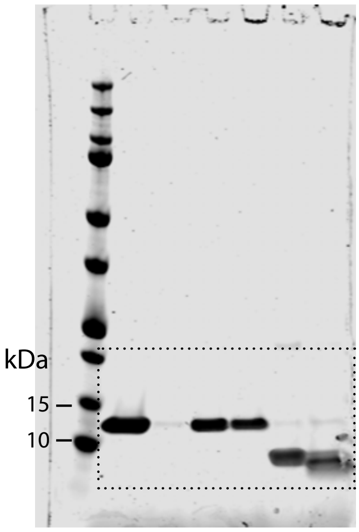

W.B. Streptavidin

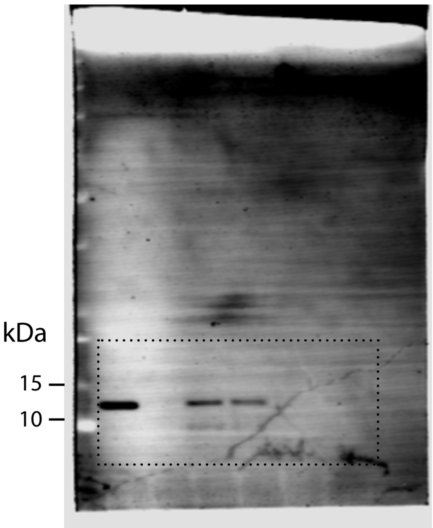

W.B. ubiquitin

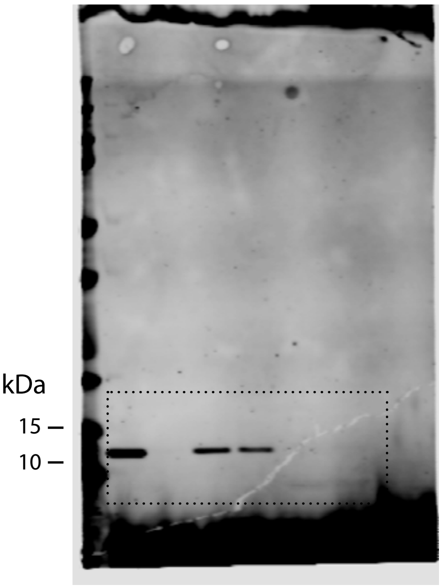

Supplementary Figure 4. Source data gels. Original source images for all data obtained by electrophoretic separation: Coomassie stained SDS-PAGE and western blots.

Figure 2A

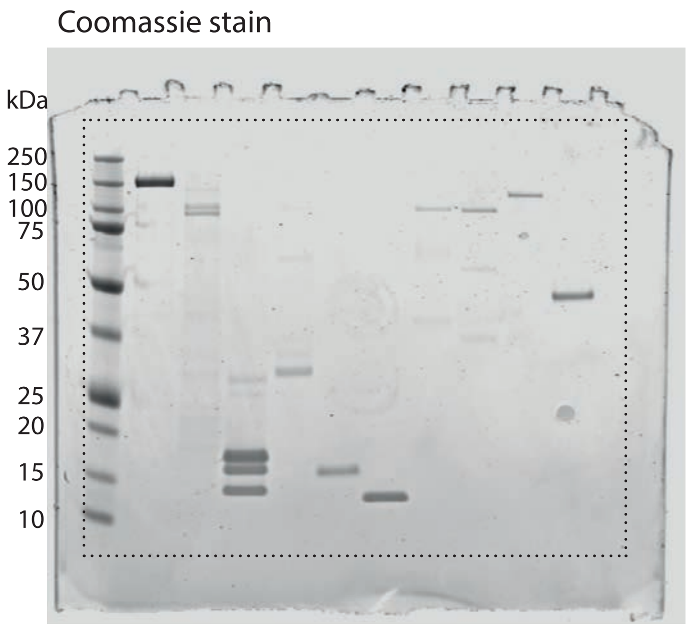

Figure 2B

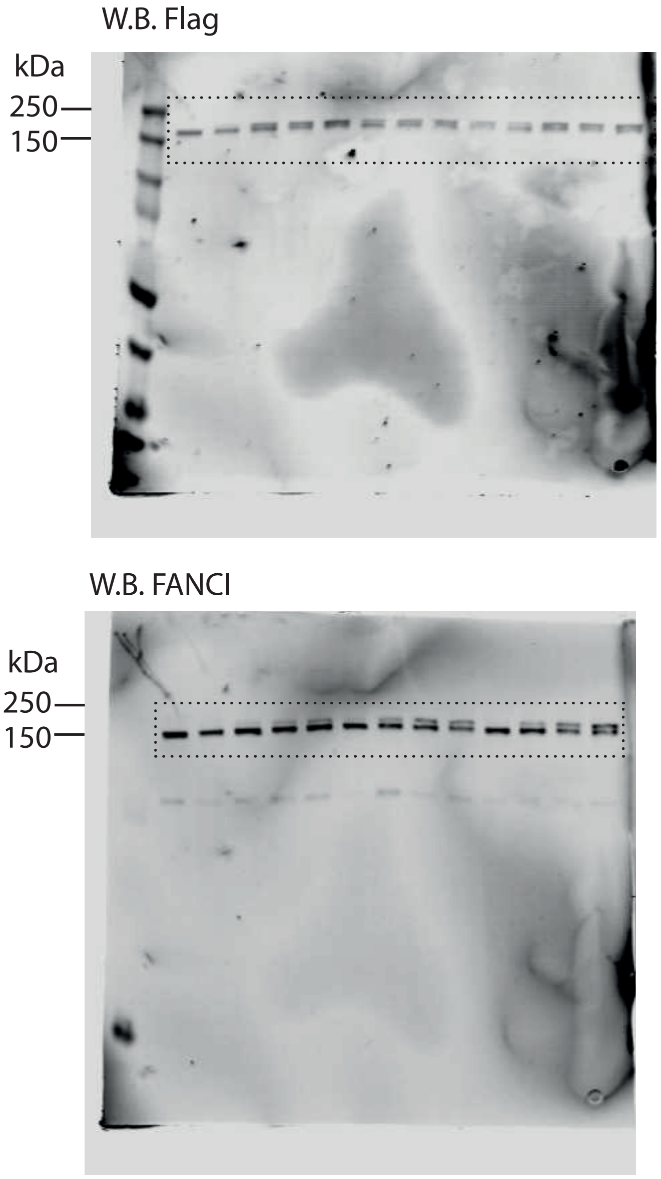

Figure 2C

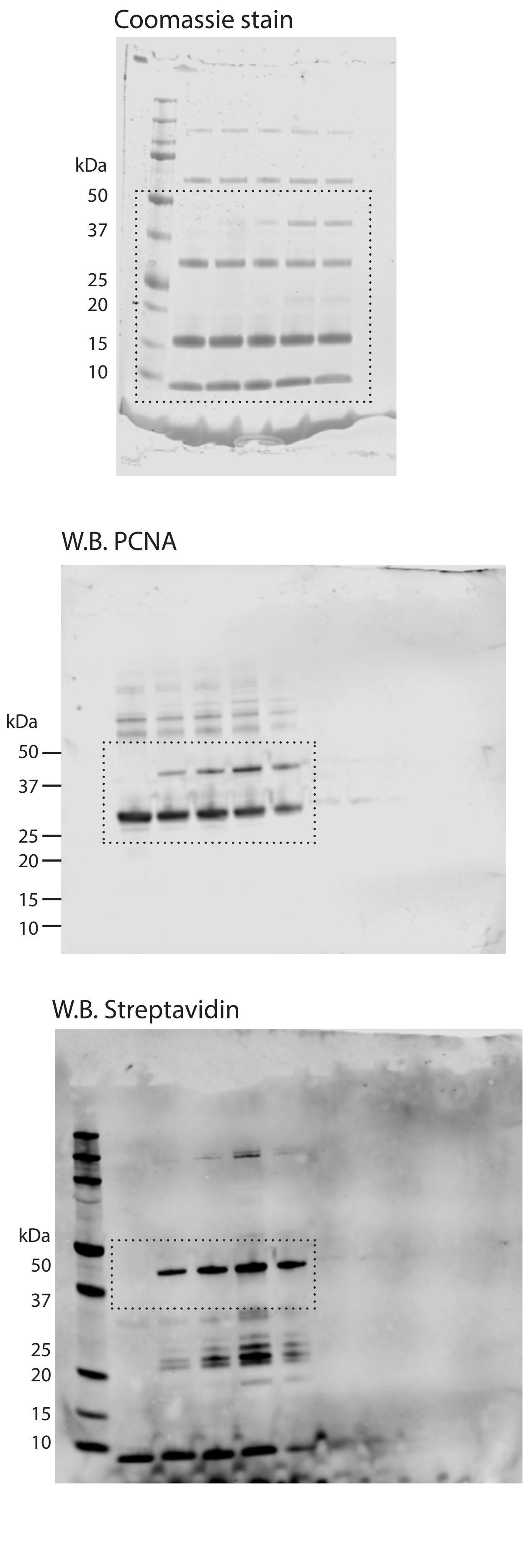

Figure 2D

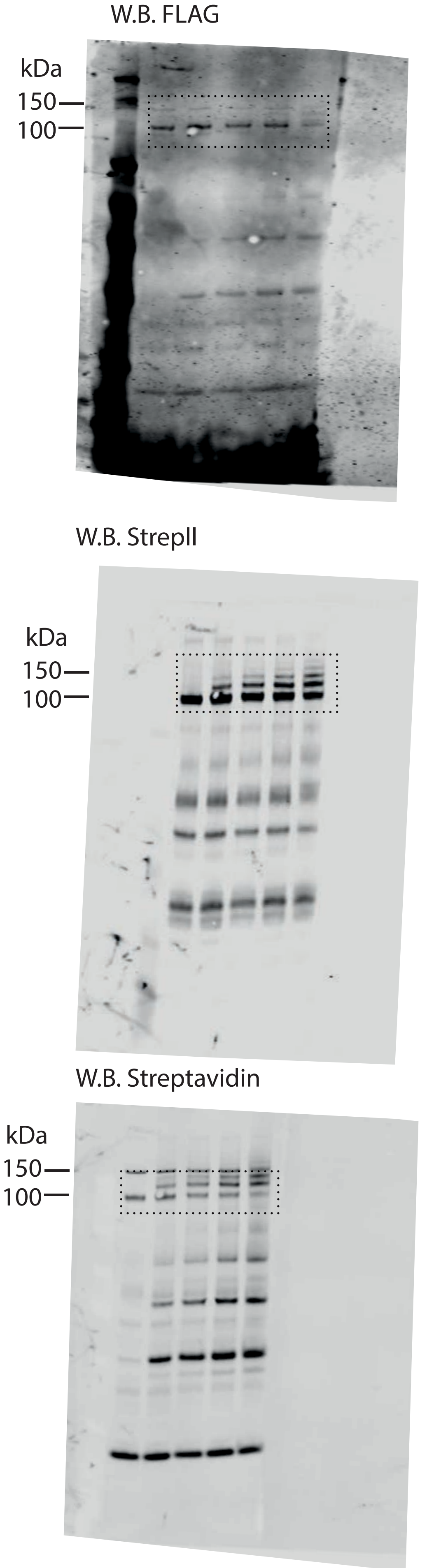

Figure 2E

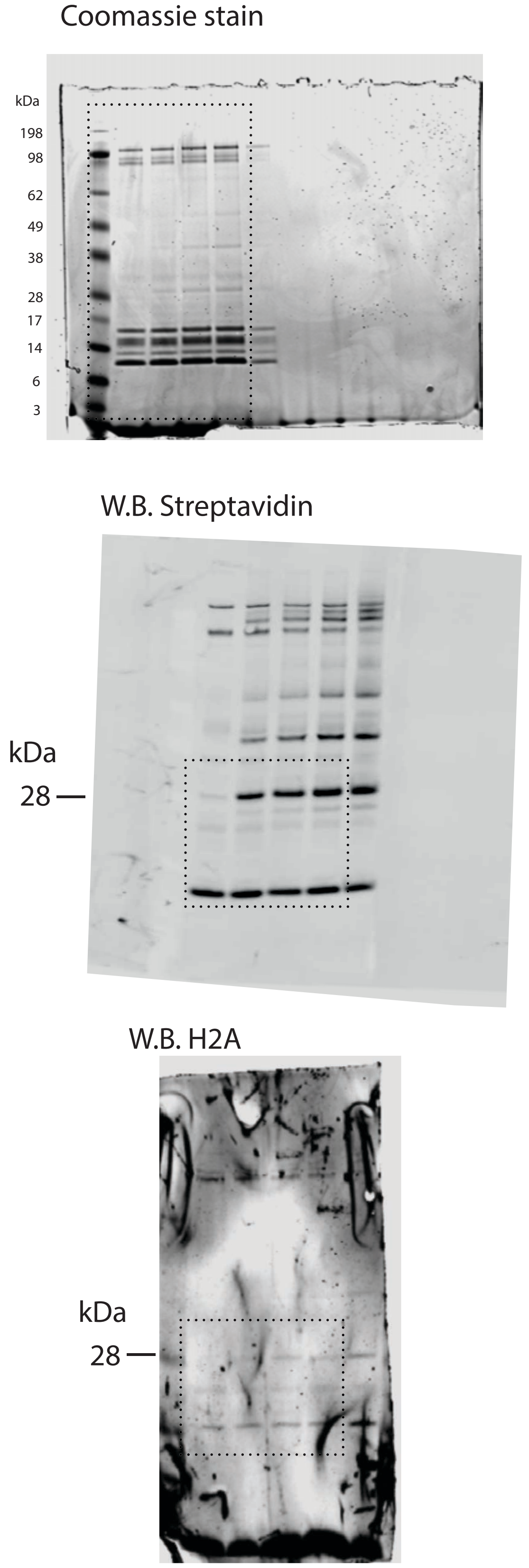

Figure 3A

Coomassie stain

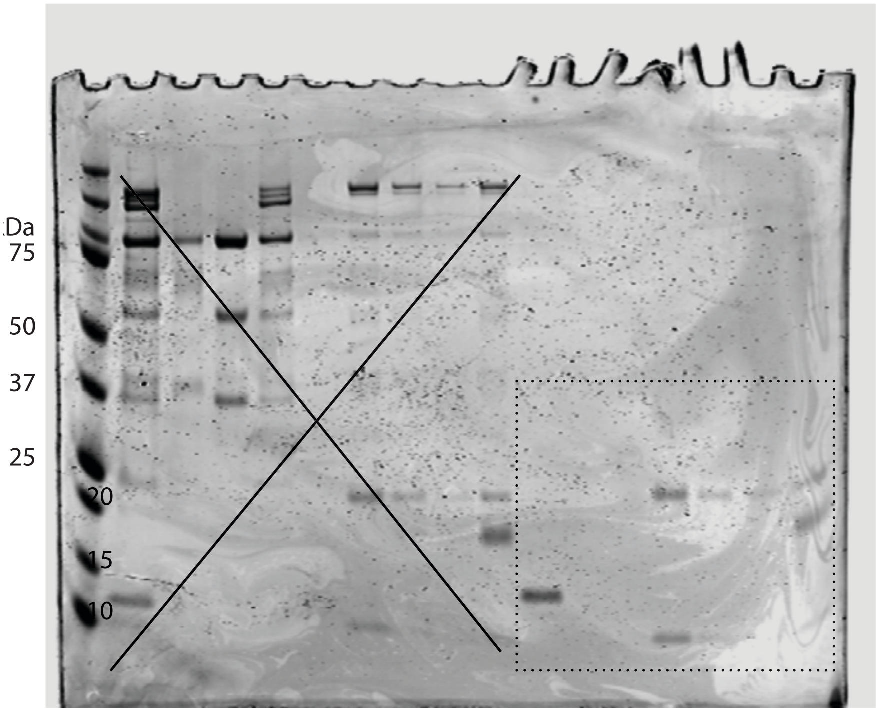

Figure 3C

Coomassie stain

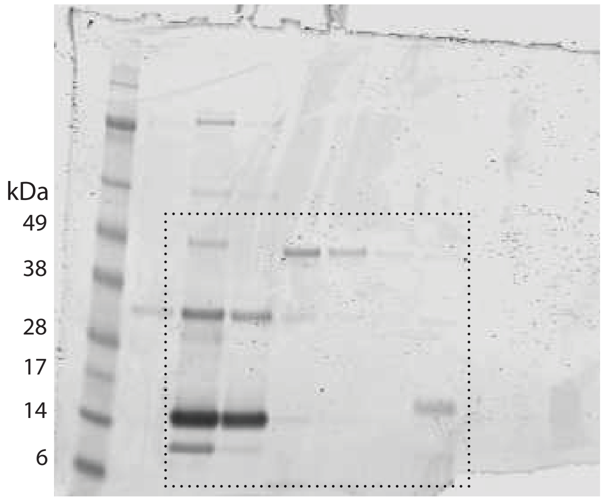

Figure 3B

Coomassie stain

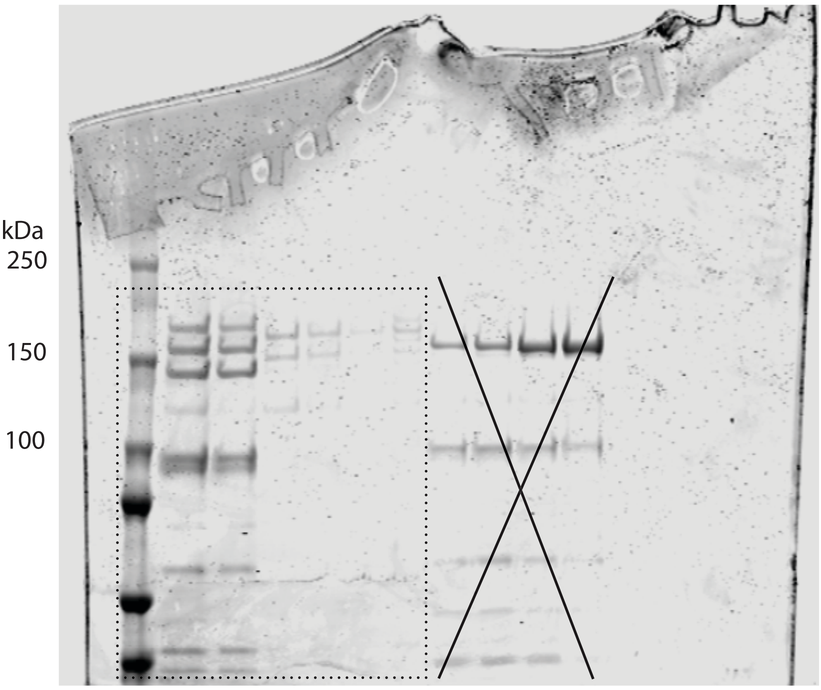

Figure 3D

Silver stain

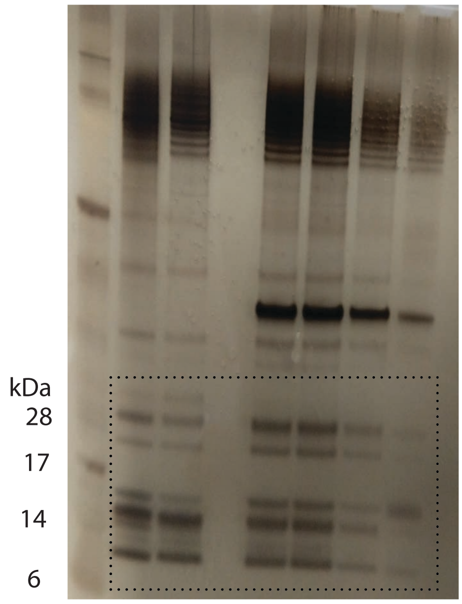

Supp. Figure 1A

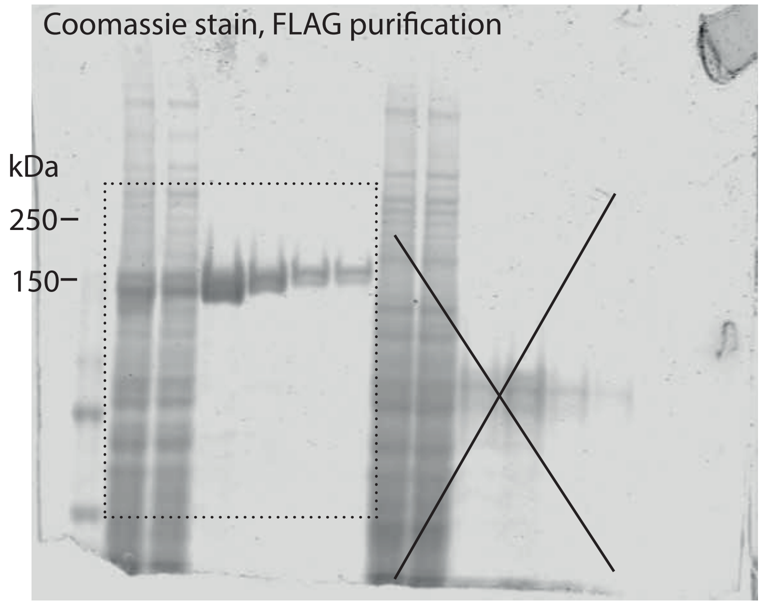

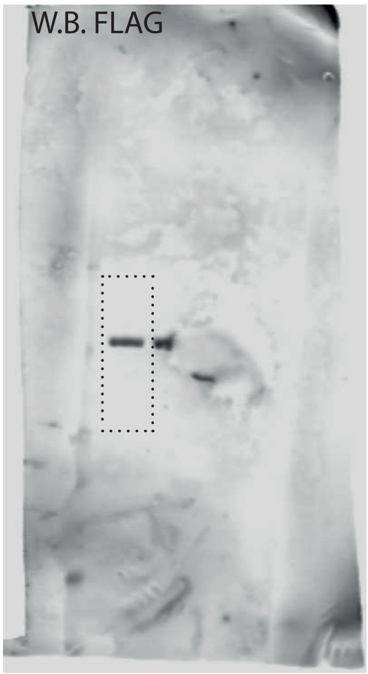

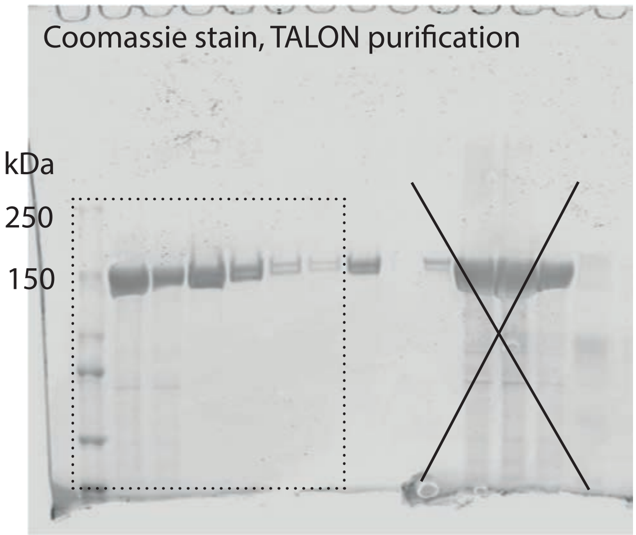

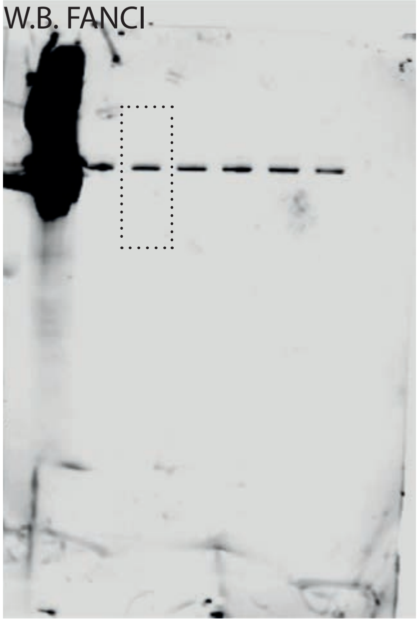

Supp. Figure 1B

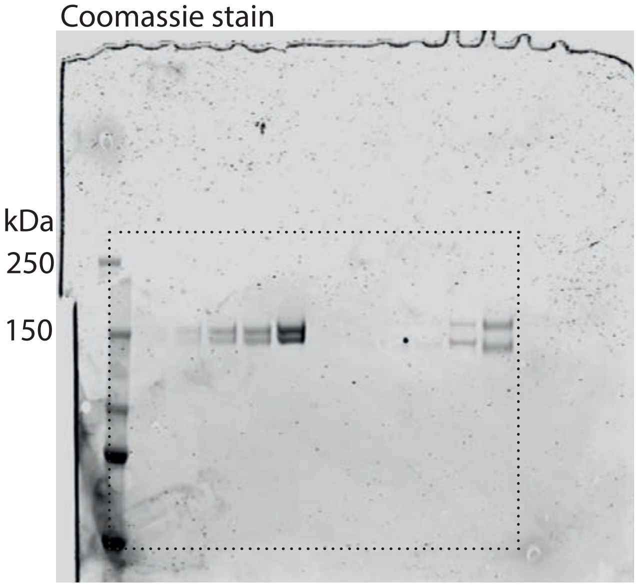

Supp. Figure 1C

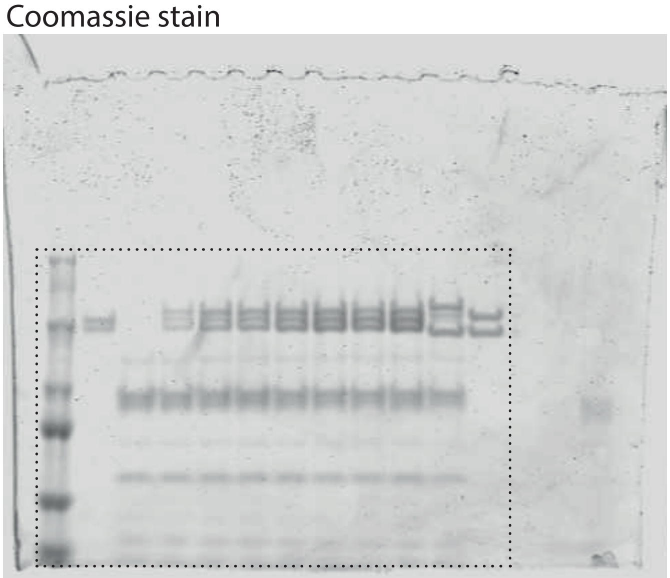

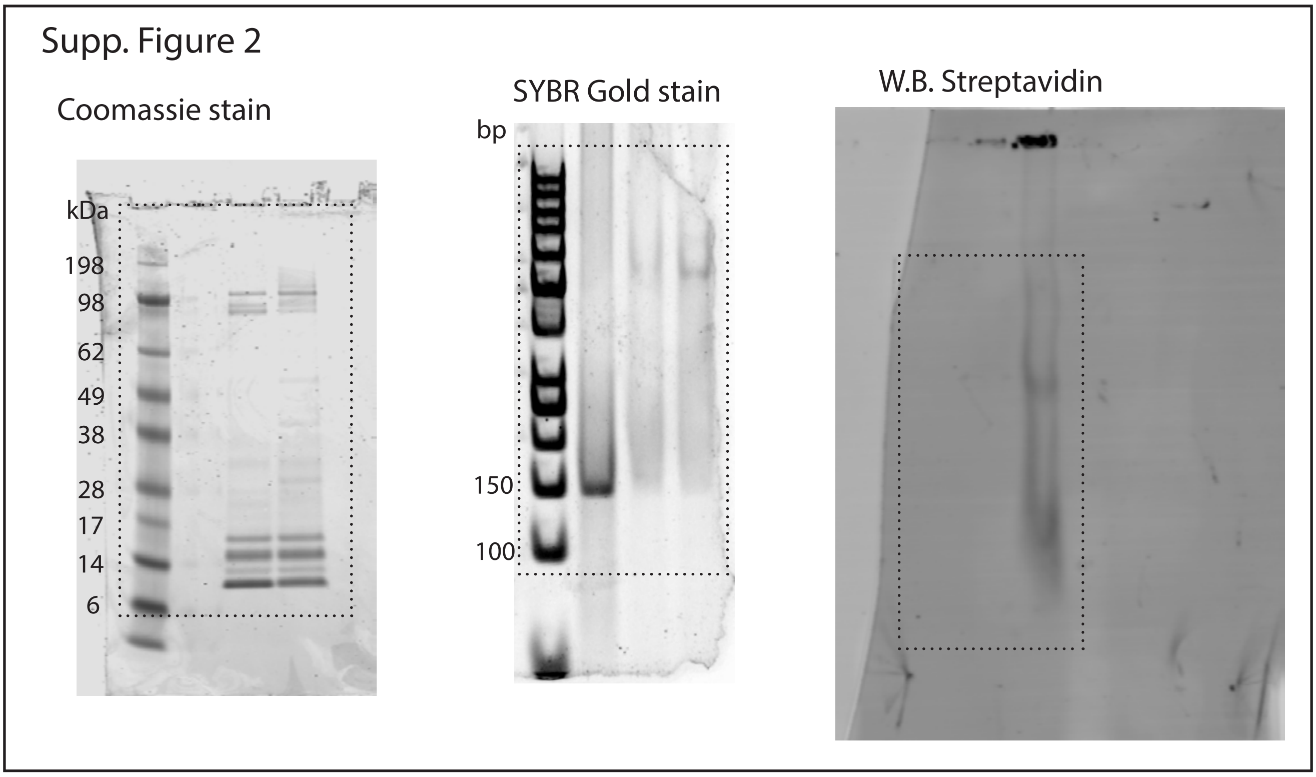
Supp. Figure 2
Coomassie stain
kDa
198
98
62
49
38
28
17
14
6
SYBR Gold stain
bp
150
100
W.B. Streptavidin
