# Supplementary material for: Preparation and purification of mono-ubiquitinated proteins using Avi-tagged ubiquitin
Source: PLoS One. 2020 Feb 24;15(2):e0229000. doi: 10.1371/journal.pone.0229000 (PMC7039436; doi:10.1371/journal.pone.0229000)
Supplement: Attachment — Submitted filename: Response to Reviewers_Tan.docx [file pone.0229000.s005.docx]

We that the reviewers for their comments and constructive feedback. Specific points are addressed below and throughout the revised manuscript.

**Reviewer #1:** In this manuscript, the authors designed ubiquitin fusion protein that can be biotinylated by BirA and can be conjugated by E3 ligases to substrates. After biotin affinity purification of modified targets, mono-ubiquitinated proteins can be purified and used for other study. This method may provide a simple strategy to study the role of ubiquitinated protein in vitro. The data support the conclusion.

Figure 1C: need to label the lane with number. This figure shows that the elution is not very efficient, have you calculated which percent of product is not eluted in the experiment?

Figure 1C is now labelled with lane number as suggested. The percentage of Avi-ubiquitin eluted is only ~50% by using 5 mM biotin, however cleavage of ubiquitin using 3C protease resulted in >95% elution of ubiquitin as indicated in Figure 1C and Figure 3A. The percentage of Avi-ubiquitin eluted is now included in the updated manuscript as requested (lines 277-281).

Figure 2A: need to label the lane with number.

Lane number is now included in Figure 2A.

Figure 2B: It is very difficult to differ FANCD2-ub and FANCD2 in the image, how the percentage was calculated?

The top panel of Figure 2B is enlarged to show bands corresponding to FANCD2-ub and FANCD2. The percentage of FANCD2-ub is calculated by quantifying the intensity of FANCD2 and FANCD2-ub bands using ImageJ, and divide the intensity of FANCD2-ub by total intensity of FANCD2.

Supple Fig 3: please indicates which line is the ubiquitylated ions in the graph.

The spectra shown in this figure correspond to the ms/ms of the b+ and y+ ions of a single peptide. Various b+ and y+ ions correspond to different fragments of the ubiquitinated peptide and together accurately identify the ubiquitinated residues indicated.

Fig 2B and 2D: please provide the images of full blot.

Images of full blot for Fig 2B and 2D are now included in Supp Fig 4.

**Reviewer #2:** The authors present a novel in vitro method for the identification of natively-mono-ubiquitinated substrates using Biotin affinity purification and apply it to the Fanconi anemia (FA)-BRCA pathway of DNA repair.

A few minor comments that should be addressed:
1) No statistics have been performed and the number of replicates remain unknown.

The number of replicates (n=3) is now added in all Figure legends as required. Similar results were achieved in triplicates.

2) The text in the body of the manuscript for Figure 1C does not correspond with the Figure. Please correct.

The text in the body of manuscript (line 259-286) is amended to correspond to the figure 1C.

3) A brief summary of the potential limitations of the Avi-ubiquitin purification protocol and the application presented in this manuscript (for example, any non-specific biotinlyation and subsequent non-specific mono-ubiquitination observed) will be helpful for scientists replicating this procedure.

A brief summary is added in the manuscript body from line 367 onwards.
